# Assessment of Local Adverse Reactions to Subcutaneous Immunoglobulin (SCIG) in Clinical Trials

**DOI:** 10.1007/s10875-017-0410-x

**Published:** 2017-07-10

**Authors:** Mark Ballow, Richard L. Wasserman, Stephen Jolles, Helen Chapel, Mel Berger, Siraj A. Misbah

**Affiliations:** 1 Department of Pediatrics, Division of Allergy & Immunology, The Johns Hopkins All Children’s Hospital, Children’s Research Institute, University of South Florida, 140 7th Ave South, St Petersburg, FL 33701 USA; 2 Medical City Children’s Hospital, Dallas, TX USA; 3 Department of Immunology, University Hospital of Wales, Cardiff, UK; 4 Oxford University Hospitals, University of Oxford, Oxford, UK; 5 CSL Behring, King of Prussia, PA USA

To the editor:

In their recent paper on the North American pivotal trial of a new 20% immunoglobulin (Ig) for subcutaneous (SC) use, Suez et al. report an incidence of local adverse events (AE) of 0.015 events/infusion and compared this to rates reported in other studies of different products [1]. Comparison of different products is inappropriate unless the products are studied contemporaneously within the same study using the same methodology, the same investigators, and the same patient populations. Judging the relative tolerability of different products accurately and determining whether manufacturing procedures, excipients, infusion supplies, pumps, and/or infusion techniques influence local site tolerability or more systemic adverse events ideally require blinded head-to-head comparisons. Crossover designs, in a targeted disease population of subjects with X-linked agammaglobulinemia and common variable immunodeficiency disorders would be preferable, and assessments should be done by the same group of investigators using a standardized grading system. In addition, consideration should be given to collecting and recording the long-term local infusion adverse events that have been described [2]. We suggest that comparing different products using different patient populations, different inclusion/exclusion criteria, and different protocols is not scientifically possible. More importantly, it is inappropriate to compare preparations and practices that have not been evaluated in the same clinical trial.

To illustrate the difficulties in making comparisons across different trials, we reviewed several SCIG studies. Borte et al. report that in an EU study of the same product as reported by Suez et al., albeit at approximately one-half the dose, the rate of local AEs was nearly five times higher, 0.069 events/infusion [3]. This is similar to another 20% SCIG preparation in an EU study [4]. In comparison, a wide range of rates of local AEs has been reported in other studies of the latter product: 0.003/infusion to 0.58/infusion (Table 1, ref. 4–6). It seems unlikely that these discrepant results reflect actual differences in the tolerability of the products in different EU and North American studies, all of which probably involved subjects predominantly of Caucasian descent with similar diagnoses. The differences may more likely reflect differences in what the subjects were taught to expect, the local effect of the volume infused, and in the methods used in evaluating, recording, and reporting AEs per se.

**Table 1:** Infusion site reactions as reported in different studies of 20% SCIG preparations

Author (reference)	Product	SCIg evaluation time points	Infusion site reaction rate/infusion	Non-aSBI systemic AEs rate	Percentage of patients discontinuing SCIG for local AEs
Suez [1]	A	Continuous as they occur^a^	0.015	0.021	0
Borte [3]	A	Continuous as they occur^b^	0.069	0.032	2.0
Jolles [4]	B	24–72 h^c^	0.056	0.034	6.5
Hagan [5]	B	24 ± 3 h^d^	0.58	0.044	4.0
Jolles [6]	B	24 ± 3 h^e^ (US)	0.524	0.16	0
Jolles [6]	B	None designated^f^ (EU)	0.001	0.09	0

^a^Recorded by the patient using an electronic diary tablet to continuously record home treatments, AEs, and additional information as they occurred. In addition, the patient was contacted by the investigator within 3–5 days after each infusion, either at the study site or at their home for follow-up to ensure appropriate documentation of AEs. The investigators reviewed patients’ eDiary entries at every site visit

^b^All patients received a diary to record home treatments, AEs, and additional information continuously as they occurred. In addition, the patient was contacted by the investigator, within 3–5 days after each infusion, either at the study site or at their home for follow-up to ensure appropriate documentation of AEs. The investigators reviewed patients’ diary entries at every site visit

^c^Local tolerability was assessed by the patients within 24 to 72 h after infusion and reviewed by the investigators during visits

^d^Local reactions were assessed by both patients and investigators. Patients assessed the overall perception of local reactions at 24 ± 3 h after the end of infusion via diaries, using a 5-point scale. Appropriate completion of the diary was monitored at every visit to the study site. Investigators evaluated local reactions (erythema, edema/induration and itching, local pain, and local heat) independently at 15–45-min post-infusion for the first four infusions at the study site and at every visit to the study site, thereafter

^e^In addition, local reactions could be reported via standard AE reporting methods at any time during the study

^f^No specific time point for assessment of local reactions was designated in the EU extension study; however, local reactions were identified manually from AE diary listings during each study visit

Despite these reported differences in tolerability, none of the studies was a single drug-related serious systemic adverse event reported. In contrast to the lack of uniformity of methods and timing of describing and recording infusion site “reactions,” all of the papers use a common well-defined efficacy endpoint, the incidence of acute serious bacterial infections, as defined in guidance documents published by the FDA [7] and EMA [8]. The rates of drug-related AEs, other than local reactions, were all within a narrow range of 0.021 to 0.16 per infusion, confirming the low frequency of systemic adverse reactions related to SCIG infusions. Finally, another indication of the tolerability of SCIG is the low percentage of subjects discontinuing treatment. Reporting of clinical trials of new SCIG products should provide more detail regarding the identification and reporting of local adverse events in the “Method” sections and should employ uniform terminology and methods for evaluating, recording, and reporting local as well as systemic infusion-related AEs. Given the current difficulties in standardizing methodologies across sites and studies, comparisons of “tolerability” of different products in reported clinical trials should be avoided.
