# Correction to: Assessment of Local Adverse Reactions to Subcutaneous Immunoglobulin (SCIG) in Clinical Trials

**DOI:** 10.1007/s10875-018-0504-0

**Published:** 2018-04-27

**Authors:** Mark Ballow, Richard L. Wasserman, Stephen Jolles, Helen Chapel, Mel Berger, Siraj A. Misbah

**Affiliations:** 1 Department of Pediatrics, Division of Allergy & Immunology, The Johns Hopkins All Children’s Hospital, Children’s Research Institute, University of South Florida, 140 7th Ave South, St Petersburg, FL 33701 USA; 2 Medical City Children’s Hospital, Dallas, TX USA; 3 Department of Immunology, University Hospital of Wales, Cardiff, UK; 4 Oxford University Hospitals, University of Oxford, Oxford, UK; 5 CSL Behring, King of Prussia, PA USA

**Correction to: J Clin Immunol (2017) 37:517–518**

**https://doi.org/10.1007/s10875-017-0410-x**

The article Assessment of Local Adverse Reactions to Subcutaneous Immunoglobulin (SCIG) in Clinical Trials, written by Mark Ballow, Richard L. Wasserman, Stephen Jolles, Helen Chapel, Mel Berger, Siraj A. Misbah, was originally published Online First without open access. After publication in volume 37, issue 6, page 517–518 the author decided to opt for Open Choice and to make the article an open access publication. Therefore, the copyright of the article has been changed to © The Author(s) 2018 and the article is forthwith distributed under the terms of the Creative Commons Attribution 4.0 International License (http://creativecommons.org/licenses/by/4.0/), which permits use, duplication, adaptation, distribution and reproduction in any medium or format, as long as you give appropriate credit to the original author(s) and the source, provide a link to the Creative Commons license, and indicate if changes were made.

